# Antibacterial Activity and Mechanism of Action of Bovine Lactoferricin Derivatives with Symmetrical Amino Acid Sequences

**DOI:** 10.3390/ijms19102951

**Published:** 2018-09-27

**Authors:** Changbao Sun, Yingying Li, Songsong Cao, Haimei Wang, Chenggang Jiang, Shiyue Pang, Muhammad Altaf Hussain, Juncai Hou

**Affiliations:** 1 Key Laboratory of Dairy Science, Northeast Agricultural University, Harbin 150030, China; sunchangbao@126.com (C.S.); 18724506316@163.com (Y.L.); Caodasong@126.com (S.C.); Wanghaimei66@126.com (H.W.); pangshiyue33@163.com (S.P.); draltafdawar@gmail.com (M.A.H.); 2 College of Food Science, Northeast Agricultural University, Harbin 150030, China; 3 Harbin Veterinary Research Institute, CAAS, Harbin 150001, China; jcg5168@163.com

**Keywords:** antibacterial peptides, bovine lactoferricin, symmetrical amino acid sequences, antibacterial activity, mechanism

## Abstract

In recent years, the overuse of antibiotics has become very serious. Many pathogenic bacteria have become resistant to them, with serious potential health consequences. Thus, it is urgent that we develop new antibiotic drugs. Antimicrobial peptides (AMPs) are important endogenous antibacterial molecules that contribute to immunity. Most have spectral antibacterial properties and do not confer drug resistance. In this paper, an 11-residue peptide (LFcinB18–28) with a sequence of KCRRWQWRMKK was modified by amino acid substitution to form a symmetrical amino acid sequence. The antibacterial activities and mechanisms of action of engineered peptides including KW-WK (KWRRWQWRRWK), FP-PF (FPRRWQWRRPF), FW-WF (FWRRWQWRRWF), and KK-KK (KKRRWQWRRKK) were investigated. The four engineered peptides could more effectively inhibit bacteria than the original peptide, LFcinB18–28. This suggested that a symmetrical amino acid sequence might enhance the antibacterial activity of AMPs. However, only peptides KW-WK, FP-PF, and KK-KK were safe; FW-WF displayed hemolytic activity. The engineered peptides shared cationic and amphipathic characteristics that facilitated interactions with the anionic microbial membranes, leading to disruption of membrane integrity and permeabilizing microbial membranes, resulting in cell death. Therefore, a symmetrical amino acid sequence and related structural parameters offer an alternative approach to the design of AMPs. This will provide a scientific basis for the design and synthesis of new AMPs.

## 1. Introduction

As multi-drug-resistant bacteria emerge, especially “superbugs,” antimicrobial peptides (AMPs) are increasingly recognized as a promising therapeutic alternative to conventional antibiotics [1]. Numerous AMPs that act as components of the innate immune system have been isolated from living organisms [2]. Currently, more than 2000 AMPs originating from natural sources have been characterized, emphasizing the importance of expanding AMPs research [3]. Antibacterial peptides have many advantages compared to antibiotics, such as a broad antibacterial spectrum, good stability, minimal side effects, and minimal drug resistance. Their principal mechanism of action involves binding to the conserved structural components of the bacterial envelope (e.g., lipopolysaccharide and lipoteichoic acid of Gram-negative and Gram-positive bacteria, respectively) [4]. This allowed AMPs to interact with the bacterial cells membrane, which was lethal to the bacteria. Some AMPs also bonded to intracellular targets and inhibited essential biological processes, including cell wall formation and the synthesis of DNA, RNA, and proteins [5]. Compared with conventional antibiotics, this mechanism could kill bacteria quickly and reduce the chances of bacteria producing resistance [6].

Bovine lactoferricin (LFcinB) is a 3.1 kDa protein and corresponds to residues 17–41 of bovine lactoferrin (LFB). The majority of these 25 amino acids are basic amino acids with isoelectric point (pI) >12. Researchers have shown that LFcinB has a broad range of antimicrobial activities against Gram-positive and Gram-negative bacteria [7,8]. It also displayed candidacidal activity [9]. Residues 19 and 36 of LFcinB are cysteines, which form a disulfide bond. While some studies had suggested that the disulfide bond was not essential for antimicrobial activities [10], several forms of LFcinB derived from peptide fragments (LFcinB17–31, LFcinB20–30, and LFcinB20–25) clearly exhibited antimicrobial activities. In fact, the antimicrobial activity was comparable to that of intact LFcinB [11]. Residues 4–9 (RRWQWR) of LFcinB have been termed the “antimicrobial center” of LFcinB, and when the C-terminal carboxyl group was amidated, the hexapeptide has been shown to display antimicrobial activity similar to full-length LFcinB [12]. Because of technical difficulties, extracting endogenous antimicrobials from organisms has low efficiency, and there was a high cost for chemical synthesis. Furthermore, the natural amino acid polypeptides are often rapidly hydrolyzed into shorter peptides as a result of enzyme action in the human body, thus greatly reducing or eliminating their antimicrobial activity [13]. Therefore, designing and screening synthetic peptides with greater stability, high antibacterial activity, and safety is needed. There are many ways to design antibacterial peptides, but the most widely used method is based on naturally occurring antimicrobial peptides that have been modified by the substitution of optimized amino acids [14].

For an antimicrobial peptide, its biological activity is closely related to many different structural parameters, including chain length, number of α-helices, hydrophilicity, cationicity, hydrophobicity, and other structural parameters [15]. In this paper, we mainly consider hydrophobicity and cationicity, due to hydrophobicity and cationicity being directly affected the affinity between AMPs and cell membrane [16,17]. We used the peptide LFcinB18–28 (KCRRWQWRMKK), which has asymmetrical amino acid sequence, as a template; the hydrophobicity amino acids included tryptophan (W), proline (P), phenylalanine (F) and the positively charged amino acids lysine (K) and arginine (R) were used to substitute the original amino acids at the N-terminus and C-terminus, making a peptide with a symmetrical amino acid sequence. Recent studies have also shown that AMPs with a symmetrical amino acid sequence have enhanced antibacterial activities [18]. The AMPs with symmetrical amino acid sequence were more likely to produce translocation to destroy the cell membrane when the AMPs interacted with the cell membrane. We engineered a total of four different AMPs, keeping the active center RRWQWR unchanged. The molecular characteristics of the four engineered peptides were greatly changed compared to the original peptide. By observing the biological activities of these peptides, we determined the effects of the symmetrical amino acid sequence and amino acid substitution. This furthered our understanding of the mechanism of bacteriostasis.

## 2. Results

### 2.1. Design and Sequence Analysis of Peptides

**Figure 1:**
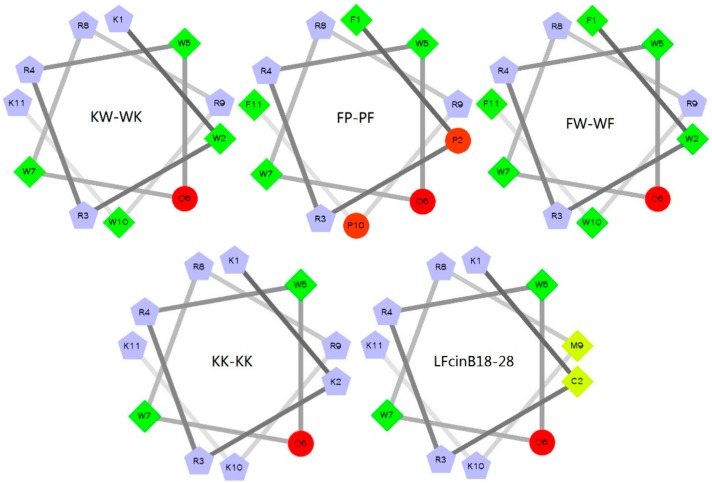
Helical wheel projections of the peptides. By default the output presents the hydrophilic residues as circles, hydrophobic residues as diamonds, and potentially positively charged as pentagons. Hydrophobicity is color-coded as well: the most hydrophobic residue is green, and the amount of green decreases proportionally to the hydrophobicity, with zero hydrophobicity coded as yellow. Hydrophilic residues are coded red, with pure red being the most hydrophilic (uncharged) residue and the amount of red decreasing proportionally to the hydrophilicity.

The physicochemical properties of the five peptides are given in Table 1. The measured molecular weights were similar to the theoretical molecular weights, suggesting that the peptides were successfully synthesized [19]. All peptides were cationic, with net charges ≥5. The net charge of peptide LFcinB18–28 was 7, the mean hydrophobicity (H) was 0.095, and the hydrophobic moment (μH) was 0.420. The H of the KW-WK, FP-PF, and FW-WF engineered peptides were 0.251, 0.478, and 0.609, respectively. The net charges were 7, 5, and 6, respectively. The μH decreased to 0.441, 0.159, and 0.054, respectively. The net charge of KK-KK was 9, but the H was reduced to −0.338 and the μH was 0.010. Using the wheel diagram (Figure 1), it was clear that, after the transformation, the box number of the peptides KW-WK, FP-PF, and FW-WF increased, which meant that the number of hydrophobic amino acid residues increased. The pentagons graphics of the peptide KK-KK was more than others, indicating that the positively charged amino acid residues increased and the net charge was large. There were yellow areas in the helix diagram for the peptide LFcinB18–28, indicating that no hydrophobic amino acid residues existed. Therefore, hydrophobicity was poor.

### 2.2. Circular Dichroism (CD) Spectra

**Figure 2:**
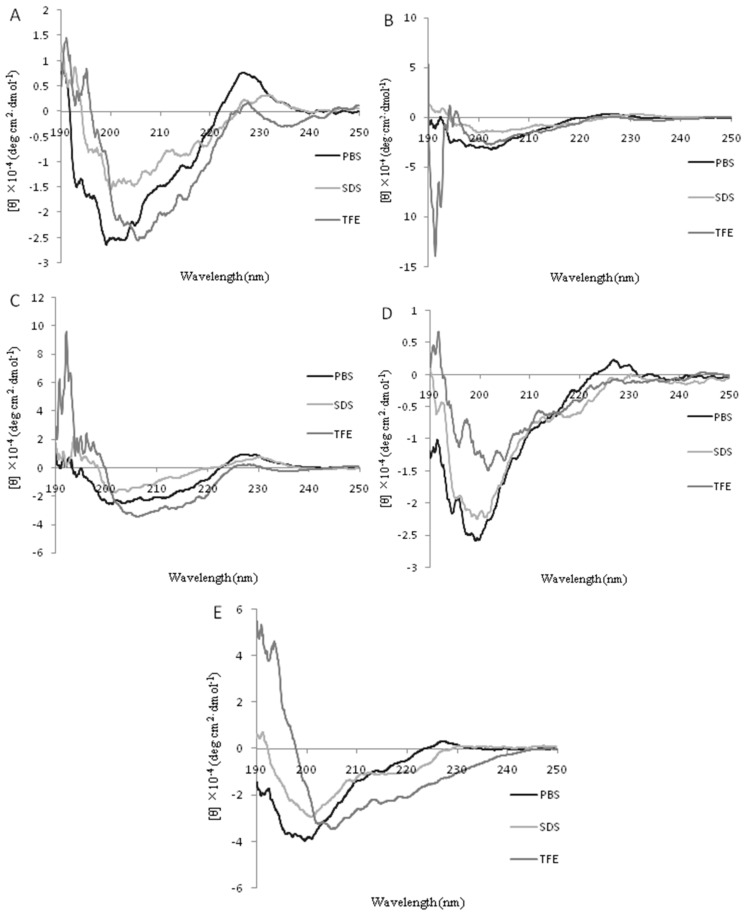
The Circular Dichroism (CD) spectrum of each peptide. The mean residue ellipticity was plotted against wavelength. The values from three scans were averaged per sample, and the peptide concentrations were fixed at 40 µM. (**A**) KW-WK; (**B**) FP-PF; (**C**) FW-WF; (**D**) KK-KK; (**E**) LFcinB18-28.

The secondary structures of the peptides in different environments (10 mM PBS, 50% TFE, and 30 mM SDS) were measured by CD spectroscopy, as shown in Figure 2. All the peptides were characteristic of unordered conformations in 10 mM PBS [20]. In 50% TFE, it was observed that there were two negative dichroic bands at 208 nm and 222 nm in the peptides KW-WK, FW-WF, and KK-KK. This was consistent with the main induction of α-helix conformations [21,22]. The peptide FP-PF did not conform to α-helix and β-sheet structures, but this needs to be studied further. The CD spectrum for LFcinB18–28 exhibited a positive peak ranging from 195 to 198 nm and a negative peak ranging from 217 to 218 nm, which reflected a β-sheet structure [23]. In the presence of 30 mM SDS (which mimics the microbial membrane environment), the spectra of the peptides KW-WK and FW-WF were the same as in 50% TFE, indicating a helical structure. Nevertheless, the spectra for the peptides FP-PF, KK-KK, and LFcinB18–28 were characteristic of disordered structures.

### 2.3. Antimicrobial Activity

The antimicrobial activities for all investigated peptides against Gram-negative and Gram-positive bacteria are summarized in Table 2. The results showed that the five peptides had the best antibacterial effects against *E. coli* ATCC25922. The comprehensive analysis of the antimicrobial activity of the peptide LFcinB18–28 was the worst. The MICs for the rest of the strains of bacteria were all more than 64 μM, except for *E. coli* ATCC25922. The geometric mean (GM) of LFcinB18–28 was 200.89 μM. Compared to the original peptide, the engineered KW-WK and FP-PF peptides had the most robust antimicrobial activities. Here, the MICs were all between 4–128 μM, and the GM was 28.00 μM and 42.22 μM, respectively. The antimicrobial activity of peptide FW-WF was also better than LFcinB18–28, but it was not greater than peptides KW-WK and FP-PF. Peptide FW-WF was effective against only a few strains, such as *Salmonella pullorum* C7913 and *S. epidermidis* ATCC 12228, and the GM was 78.67 μM. The peptide KK-KK was not effective in comparison to other engineered peptides. The bacteriostatic effect was not improved significantly, and the GM was163.55 μM.

### 2.4. Hemolytic Activity

**Figure 3:**
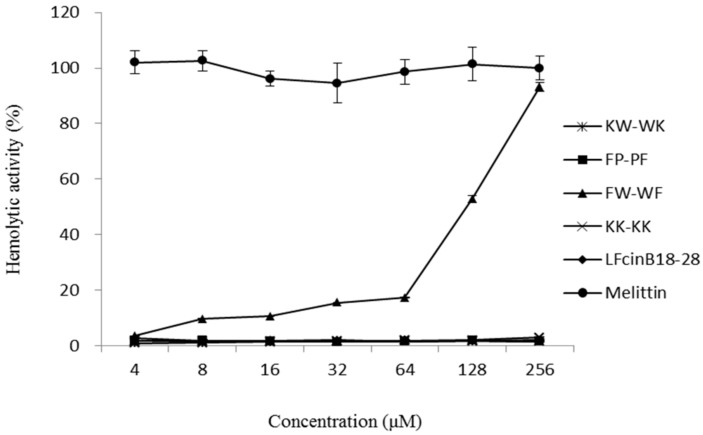
Hemolytic activity curves of each peptide against human red blood cells.

The hemolytic activities of the AMPs were evaluated by quantifying their ability to lyse human erythrocytes (Figure 3). When the peptides concentrations were between 4–256 μM, the hemolytic activity of peptides KW-WK, FP-PF, KK-KK, and LFcinB18–28 was significantly lower than that of melittin (*p* < 0.05). The hemolytic activities were less than 20% when the peptide concentrations were between 4–64 μM. The hemolytic activity (52.91%) of FW-WF was significantly higher than the other four peptides at a concentration of 128 μM (*p* < 0.05). However, compared to melittin, the hemolytic activity was still relatively low.

### 2.5. Cytotoxicity

**Figure 4:**
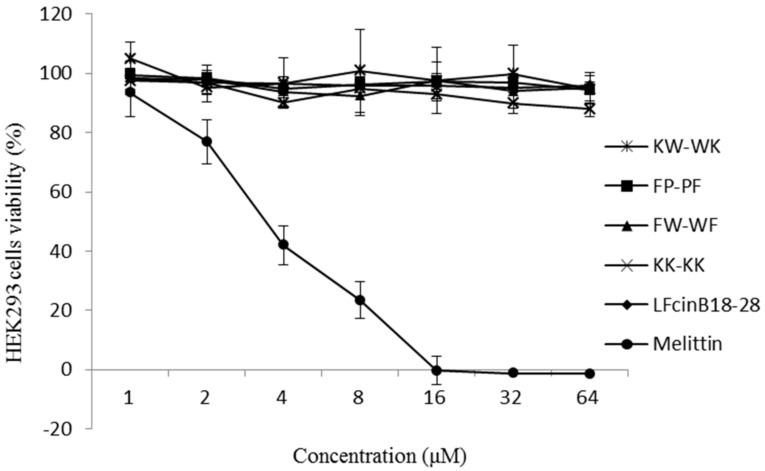
Cytotoxicity of each peptide against HEK293 cells.

The cytotoxicity of the peptides was determined using HEK293 cells, as shown in Figure 4. The five peptides had almost no cytotoxic activity at concentrations of 1–64 μM, and the cells’ viability was very high. In contrast, the peptide melittin exhibited greater cytotoxic activity, and the cell viability was only 41.95%, 23.40%, and 0% at concentrations of 4, 8, and 16 μM, respectively.

### 2.6. Stability

To determine peptide stability, the antimicrobial activity of each peptide was tested following exposure to physiological concentrations of various salts, high temperature, and proteases. Table 3 showed the MICs of the peptides after treatment with seven different cations. K^+^ and Fe^3+^ did not affect the activity of KW-WK, and the MIC was still 4 μM after treatment. However, other cations changed the MICs to 8 μM, which was slightly higher than that 4 μM. However, these peptides still maintained high levels of antimicrobial activity in these conditions. Na^+^ had a significant influence on the antimicrobial activity of FP-PF. The MIC was 64 μM after the treatment. The antimicrobial activity of peptide FW-WF was not affected by K^+^, Mg^2+^, Zn^2+^, Fe^3+^, or NH_4_^+^. There were six cations that enhanced the antimicrobial activity of KK-KK. However, Mg^2+^ reduced this peptide’s antimicrobial activity. Zn^2+^, Fe^3+^, and NH_4_^+^ enhanced the antimicrobial activity of peptide LFcinB18–28.

Table 4 shows the results of the antimicrobial activity assay following heat treatment of 100 °C for 1 h. The MICs of peptides KW-WK and LFcinB18–28 did not change, which suggested that the thermal stability of these peptides was high. The MICs of peptides FP-PF and FW-WF changed from 4 μM to 8 μM, and heat treatment had a small influence on the effects of the two peptides. Interestingly, heat treatment completely eliminated the antimicrobial activity of peptide KK-KK.

The MIC values for the antimicrobial peptides in the presence of proteolytic enzymes were shown in Table 5. Peptide KW-WK was completely inactivated by trypsin. The MICs of KW-WK were 16 μM, 8 μM, and 8 μM following treatment with pepsin, papain, and protease K, respectively. Although this was increased, it still had better antimicrobial activity. Papain and protease K had little influence on the antimicrobial activities of FP-PF and FW-WF. However, trypsin and pepsin had a significant influence on them. The four proteases significantly influenced the antimicrobial activity of peptides KK-KK and LFcinB18–28.

### 2.7. Mechanism of Action of the Peptides

The peptide FW-WF displayed hemolytic effects in hemolytic activity assays; therefore, only the peptides KW-WK, FP-PF, and KK-KK were investigated in the study of mechanism assays.

#### 2.7.1. Outer Membrane Permeability

**Figure 5:**
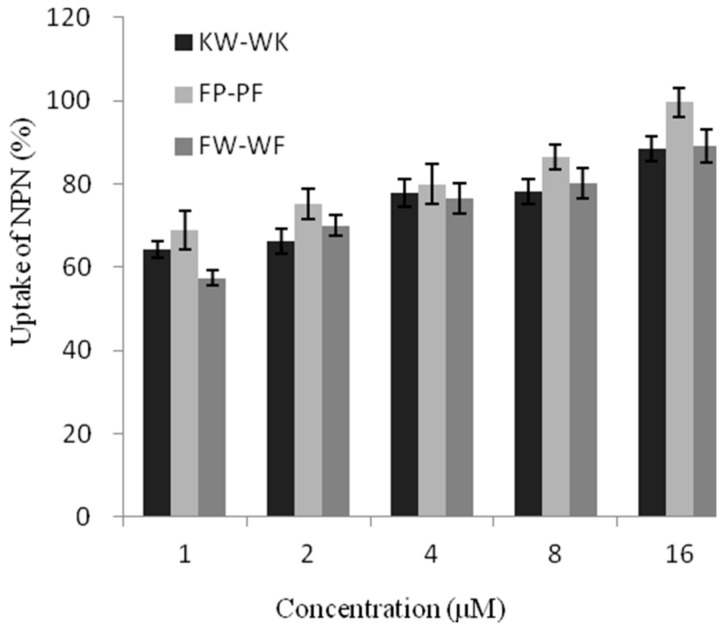
Uptake of NPN in *E. coli* UB1005 cells treated with different concentrations of antimicrobial peptides.

The peptides were evaluated for their abilities to permeabilize the bacterial outer membrane in a dose-dependent manner (0–16 μM) in Ethyleneimine (*N*-Phenyl-1-Naphthy Lamine, NPN) uptake assays (Figure 5). Peptides KW-WK, FP-PF, and FW-WF affected the permeability of the outer membrane. At 1 μM, the outer membrane permeability of the engineered peptides was over 50%. At this concentration, peptide FP-PF induced a greater uptake of NPN compared to peptides KW-WK and FW-WF, which suggested that the damage to the outer membrane was greatest after treatment with peptide FP-PF.

#### 2.7.2. Inner Membrane Permeability

**Figure 6:**
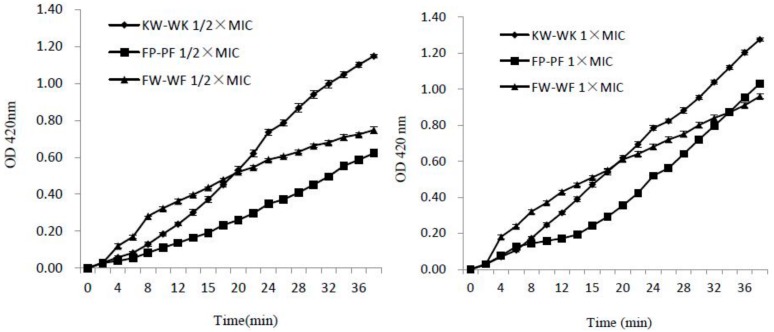
Release of cytoplasmic β-galactosidase in *E. coli* UB1005 cells treated with antimicrobial peptides at concentrations of 1/2× MIC and 1× MIC.

To further investigate the ability of the peptides to permeabilize the bacterial inner cell membrane, we used the *O*-Nitrophenyl-β-d–galacto pyranoside (ONPG) assay. As shown in Figure 6, three peptides induced rapid increase in the permeability of the inner membrane at 1× MIC and 1/2× MIC within 38 min, and these peptides maintained a consistently increasing trend. The inner membrane permeability of the peptides at 1× MIC was higher than that at 1/2× MIC, which meant that the release was dose-dependent. Compared with peptides KW-WK and FP-PF, FW-WF was not as effective at disrupting the integrity of the inner cell membrane.

#### 2.7.3. Cytoplasmic Membrane Electrical Potential

**Figure 7:**
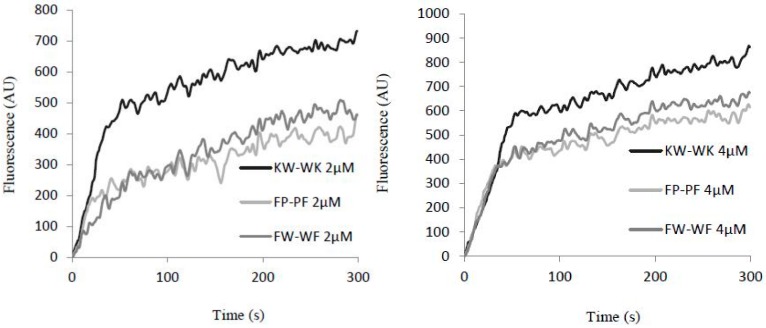
Cytoplasmic membrane depolarization of *E. coli* UB1005 follow treatment with the antimicrobial peptides at concentrations of 2 μM and 4 μM assessed by release of the membrane potential-sensitive dye diSC3-5.

When the cell membrane was disrupted, diSC3-5 dye enters the cells, which could be assayed by measuring the intensity of fluorescence. Cell membrane depolarization was greatest following treatment with increasing concentrations and incubation times for peptide KW-WK, followed by FW-WF and then FP-PF (Figure 7). The effect of these three peptides on cell membrane depolarization increased gradually in *E. coli* UB1005. At the same concentration and increasing reaction time, the fluorescence intensity increased gradually, indicating that cell depolarization was time-dependent. At different concentrations, each peptide showed different expression. When concentrations increased, the fluorescence intensity increased accordingly, suggesting that the extent of cell depolarization was also concentration-dependent.

#### 2.7.4. Flow Cytometry

**Figure 8:**
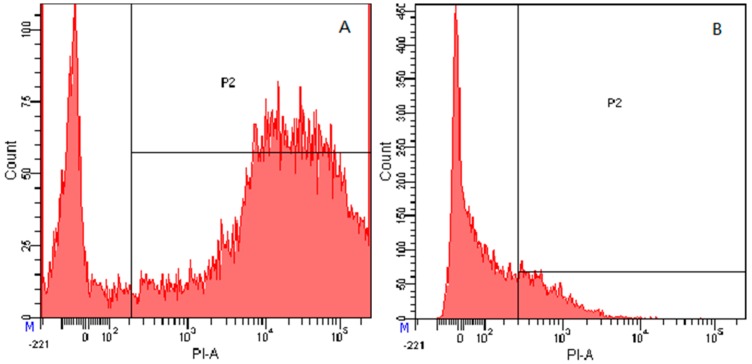

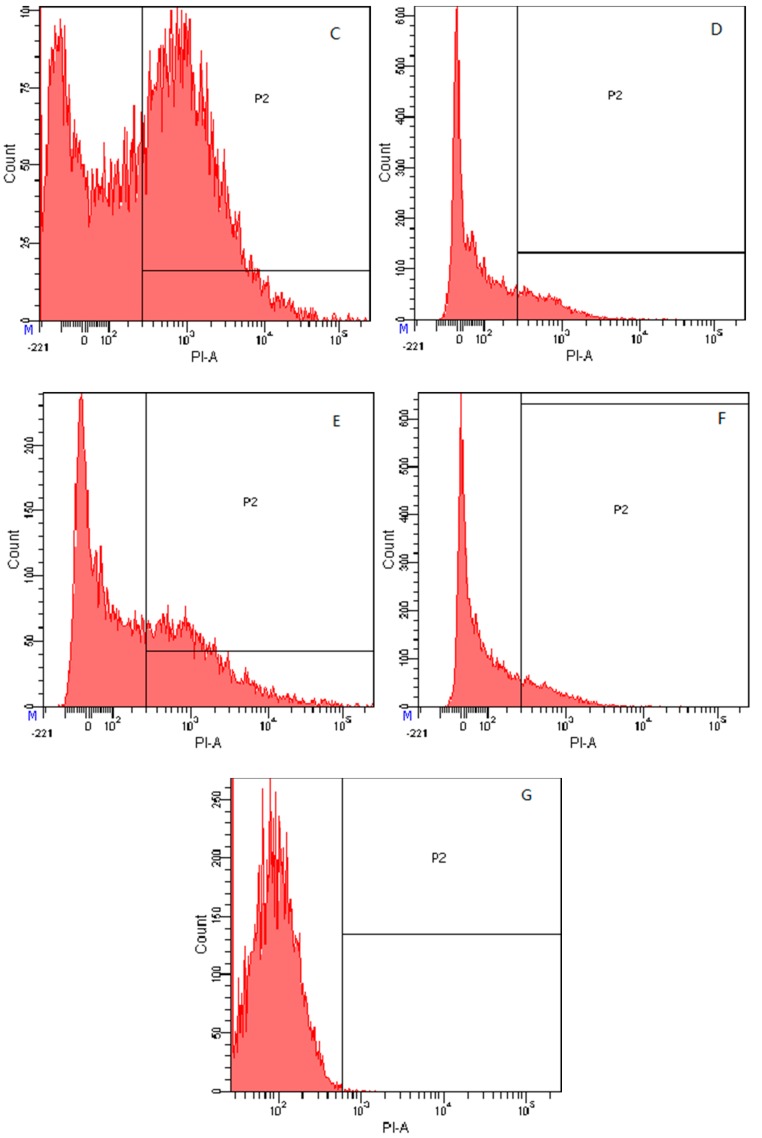
Membrane damage in *E. coli* ATCC25922 cells treated with the peptides as measured by an increase in fluorescence intensity of PI. (**A**) KW-WK 1× MIC; (**B**) KW-WK 1/2× MIC; (**C**) FP-PF 1× MIC; (**D**) FP-PF 1/2× MIC; (**E**) FW-WF 1× MIC; (**F**) FW-WF 1/2× MIC; (**G**) the control processed without peptides.

To further study the antimicrobial effects of the peptides on microbial cells, the integrity of the bacterial cells membrane was observed following treatment with these peptides. Propidium iodide (PI) was used to fluorescently label nucleic acids in the bacterial cells when the cytoplasmic membrane integrity was disrupted. As shown in Figure 8G, in the absence of the peptides, 99.8% of the cells displayed no PI fluorescent signal, indicating that the cells membrane were intact. However, following treatment with the peptides in 1× MIC (Figure 8A,C,E) and 1/2× MIC (Figure 8B,D,F), the cells became labelled with PI, and the intensity of staining correlated with increasing concentrations of the peptides. The fluorescence detection results for the 1/2× MIC concentration of peptides KW-WK, FP-PF, and FW-WF showed that the survival rates of the cells were 76.3%, 80.3%, and 82.5%, respectively. For the 1× MIC concentration, the rates were 23.6%, 44.8%, and 58.7%, which showed that the effect of AMPs on cells membrane integrity was concentration-dependent.

#### 2.7.5. Electron Microscopic Studies

**Figure 9:**
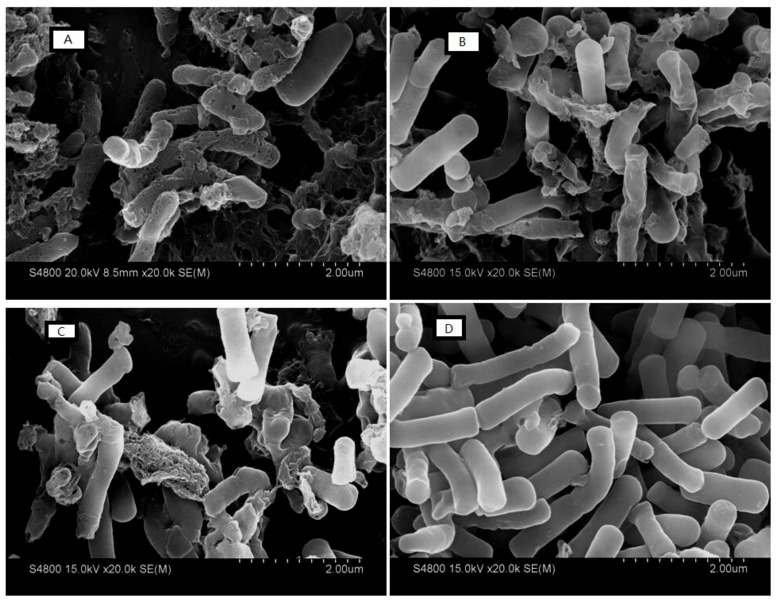
SEM micrographs of *E. coli* ATCC25922 cells treated with the antimicrobial peptides. (**A**) KW-WK; (**B**) FP-PF; (**C**) FW-WF; (**D**) the control was processed without peptides.

Morphological characteristics of the bacterial cells surface of *E. coli* ATCC25922 were observed by SEM before and after treatment with the peptides (Figure 9). Untreated cells exhibited an intact and smooth surface, without any visible holes in the membrane or cellular debris (Figure 9D), indicating that the bacteria were in good condition. Following treatment with peptides for 2 h at the concentration of 1× MIC (Figure 9A–C), the shape of the *E. coli* ATCC25922 cells was perturbed and the cells were broken. The contents of the bacteria leaked out, and the cells appeared wrinkled. The surface morphology of the cells was different after treatment with different AMPs, which indicated that the AMPs lysed the cells in diverse ways. Figure 9A showed the cells’ state after treatment with peptide KW-WK. Compared to Figure 9B and Figure 9C, the cell surface was seriously damaged. Most of the cells were destroyed, and pores appeared in the membrane. The cells were crushed and completely cracked. After treatment with peptides FP-PF and FW-WF, the cells were partly destroyed, and the intact structure of *E. coli* ATCC25922 could be seen in the field of view. The extent of cell disruption was less than that following treatment with peptide KW-WK.

**Figure 10:**
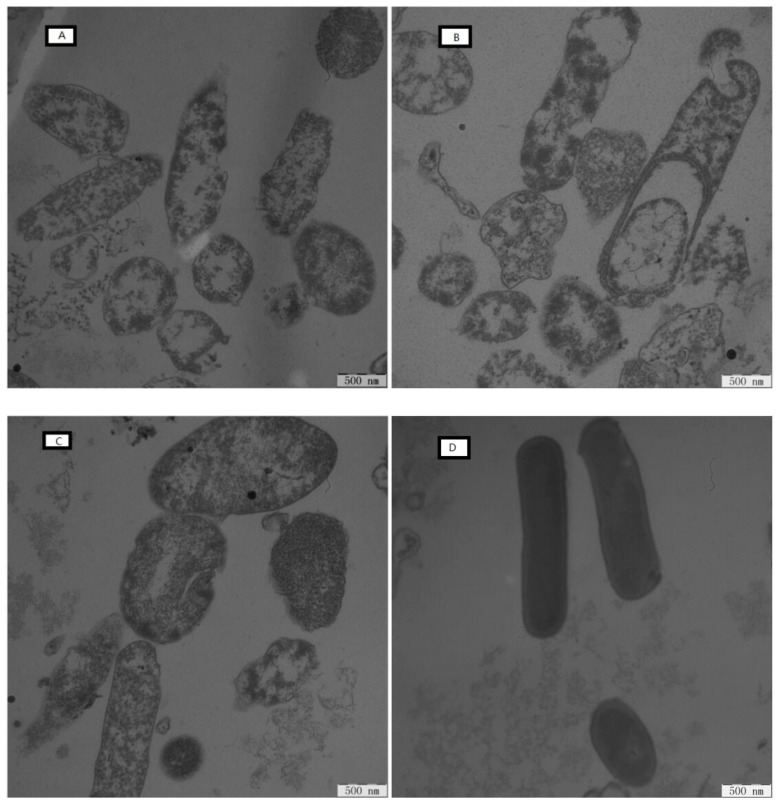
TEM micrographs of *E. coli* ATCC 25922 cells treated with the antimicrobial peptides. (**A**) KW-WK; (**B**) FP-PF; (**C**) FW-WF; (**D**) the control was processed without peptides.

The ultrastructural features of the *E. coli* ATCC25922 cells were observed using TEM. Control cells exhibited a smooth, intact membrane surface and dense internal structure (Figure 10D). However, the shape of the cells was disrupted after 2 h of treatment with peptides at the concentration of 1× MIC (Figure 10A–C). Severe damage was observed, and the cell membrane had gaps. The distribution of intracellular contents was not uniform. Different peptides elicited different degrees of antibacterial damage. Figure 10A showed the cells’ state after treatment with peptide KW-WK. Compared with Figure 10B,C, the cell membrane was completely ruptured, the edges were not clear, the contents of the cell were diminished, and there were many gaps in the membrane. Although the contents were lost after treatment with peptides FP-PF and FW-WF, this was not serious, especially for treatment with peptide FW-WF, where the cell edge was still clear and the cell membrane rupture was incomplete.

## 3. Discussion

LFcinB has a broad spectrum of activity against Gram-negative and Gram-positive bacteria [9]. Because of a unique bacteriostatic mechanism, it significantly reduces the likelihood of bacterial resistance [24]. However, extracting natural LFcinB from organisms is expensive, and it is unstable in many circumstances. The design and modification of AMPs can solve this problem. The sequential template method means that a natural antimicrobial peptide can be studied by inserting selected amino acid in the peptide sequence to alter the net positive charge, in addition to other features, such as α-helices, hydrophilicity and hydrophobicity. Antimicrobial peptides with more robust activity and a wider antimicrobial spectrum can be obtained. Many of the physiological activities associated with LFcinB have been localized to the cationic N-terminal lobe of this iron-binding protein [9]. The peptide LFcinB18–28 is an 11-residue peptide with the sequence KCRRWQWRMKK. It contains the active center RRWQWR [12]. We used the method of amino acid substitution to obtain four engineered peptides with symmetrical amino acid sequences.

Our results indicated that the structure of the three engineered peptides (KW-WK, FW-WF, and KK-KK) in the simulated membrane environment would be transformed into an α-helical structure. Studies have shown that an α-helical structure can enhance the activity of antimicrobial peptides because the structure of the water-lipid is favorable to the combination and penetration of bacterial cell membranes by antimicrobial peptides [25]. This may explain why the antimicrobial activity of these peptides was higher than that of LFcinB18–28. Peptide FP-PF had an irregular structure in SDS, probably because it contained proline. Studies have shown that the extent of helicity decreases as the number of proline residues increases [26]. The original LFcinB18–28 peptide was a β-fold structure. This is likely because the amino acid sequence contained a cysteine, which is a necessary amino acid for forming the β-fold structure [27].

The antimicrobial assays proved that engineered peptides were much more active than LFcinB18–28. This showed that the symmetrical amino acid sequence could improve the antimicrobial activities of AMPs. The transformation of LFcinB18–28 was successful. When we analyzed the molecular characteristics of each peptide, we found that the results were consistent with previous findings that the hydrophobicity of KW-WK, FP-PF and FW-WF was enhanced compared with LFcinB18–28, suggesting that hydrophobic amino acid substitutions increased the hydrophobicity of AMPs and, thus, increased the antimicrobial activity [28]. Hydrophobic groups enabled the peptide chains to form aggregates in solution via hydrophobic interactions, increasing the affinity for eukaryotic membranes [17]. The ability of AMPs to form an α-helix was also strengthened with enhanced antimicrobial activity [29]. However, the increased hydrophobicity was not necessarily better. If hydrophobicity was too high, this would result in self-aggregation and precipitation, reducing the antimicrobial activity of AMPs [30]. The hydrophobic moment represented the cumulative hydrophobicity of a peptide and reflected the structural characteristics of the interaction between peptides and membranes. It could be used to express the hydrophilicity of AMPs. The higher the hydrophobic moment, the stronger the ability of AMPs to break the membrane [31]. Cationic properties are essential for most AMPs with antimicrobial activity because positive charge promotes the activity of antimicrobial peptides at low concentrations to enrich the surface of the film to kill bacteria. This directly affected antimicrobial activity [32]. The net charge and hydrophobic moment of peptide KW-WK were relatively higher, which enabled it to have better bacteriostasis than FP-PF and FW-WF, even though they were more hydrophobic than peptide KW-WK. The net charge of peptide KK-KK was 9, but the inhibition was still very weak. These results indicated that if the net charge was too high, bacteriostasis would be restricted [16]. This may be because too much positive charge can enable the AMPs to firmly bind the head of the phospholipid. Thus, the film-penetrating efficiency is reduced. At the same time, when the number of positive charges exceeds a certain critical value, this leads to electrostatic repulsion between the AMPs, which is stronger than their electrostatic attraction to the membrane. This prevents the accumulation of AMPs molecules and the formation of transmembrane pores, reducing membrane lysis and antimicrobial activity [33]. Therefore, the antimicrobial activities of AMPs were likely related to hydrophobicity, hydrophobic moment, and net charge.

The hemolytic activity of the peptides against human erythrocytes and the cytotoxicity of the peptides against HEK 293 cells have been suggested to be major parameters determining peptide toxicity in higher eukaryotic cells. Melittin is one of the most biologically active polypeptides, accounting for 50% of the dry weight of bee venom. It has high hemolytic and cytotoxic effects, and is commonly used as a control peptide [34]. Compared with melittin, we observed in this study that the hemolytic activity and cytotoxicity of the five peptides were not significant. Compared with the other four peptides, FW-WF showed a slightly higher hemolytic effect, probably because both ends of the peptide contained the hydrophobic amino acids tryptophan and phenylalanine, which increased the overall hydrophobicity [35]. Frecer et al. [36] also showed that hydrophobic amino acids affected hemolytic. The treatment index (TI) was defined as the ratio of MHC to MIC, showing antimicrobial specificity of antimicrobials, the larger the value of TI, the greater the antimicrobial specificity [37]. As shown in Table 2, the cell selectivity of peptides KW-WK and FP-PF was better and safer. Thus, they can be widely used in the fields of food, medicine, and feed.

If AMPs become widely used in production, they will be subject to a variety of destabilizing factors, such as high-temperature environments, many kinds of proteases, and a variety of cations [38]. Therefore, it is necessary to determine the stability of each peptide. Interestingly, when cations with physiological concentrations were added to the medium, only a few cations had a slightly inhibitory effect on specific peptides. Some inhibitory effects were even increased. It is believed that the electrostatic interactions between the cationic portions of the peptide and the negative charge on the surface of the bacteria facilitated the interaction between the lipophilic regions of the peptide with the cell membrane. This led to the destruction of the cell membrane, which caused the cells to die. Generally, most cationic AMPs are salt-sensitive, displaying reduced or absent antimicrobial activity at high salt concentrations. However, at relatively low physiological concentrations, this is not always the case. At high concentrations, divalent cations progressively increased membrane rigidity through electrostatic interactions with negatively charged phospholipids, which slowly hinders pore formation [34]. Previous studies established that the antimicrobial activity of LFcinB was modulated by the presence of monovalent and divalent cations [39]. Na^+^ reduced the antimicrobial activity of the peptide FP-PF. This may because cations affected the activity of AMPs by interfering with their ability to bind membranes [40]. The fact that small amounts of divalent cations can contribute to peptide membrane binding could explain why NH_4_^+^ increased the activity of FW-WF. Fe^3+^ increased the activity of KK-KK and LFcinB. After heating at 100 °C for 1 h, we found that the antimicrobial activity of the peptides was still high, except for peptide KK-KK. This was consistent with a previous report that most AMPs were thermally stable [41]. After treatment with four different proteases, it was found that the effects of these proteases were diverse. Only trypsin significantly decreased the antimicrobial activity of the peptides. Research has shown that the cleavage sites of trypsin involve the peptide bond formed by lysine and arginine [42], which the five AMPs all contained. Thus, their activities were inactivated. Papain is a sulfhydryl protease, and its cleavage site is the peptide bond formed by arginine, lysine and glycine, which peptide KK-KK has. Thus, it was unstable after treatment with papain.

Based on the antimicrobial activity test for these antimicrobial peptides, we knew that peptides KW-WK, FP-PF and FW-WF had strong antimicrobial effects. Therefore, we studied their bacteriostatic mechanism. Phosphatidyl glycerol, cardiolipin and phosphatidylserine are predominant components of the bacterial membrane [43]. Usually, in the first step of bacteriostasis, cationic peptides are selectively combined with negatively charged composition of the outer membrane, such as lipopolysaccharide [44,45]. Then, these peptides are readjusted and inserted into the cytoplasmic membrane lipid bilayer, causing the disruption of membrane permeabilization and integrity or pore/ion channel formation. At the same time, the membrane potential disappears [46]. In this study, we found that three peptides (KW-WK, FP-PF, and FW-WF) had a concentration-dependent effect on the permeabilization of the outer membrane of *E. coli* UB1005. All peptides had the ability to permeabilize the inner membrane to ONPG at 1× MIC and 1/2× MIC, which was indicative of strong, concentration-dependent membrane permeabilizing ability. From the flow cytometric analysis, we found that all peptides killed bacteria by damaging cytoplasmic membrane integrity. Our SEM and TEM results further confirmed that all peptides had potent interactions with the membrane structure, disrupted the cell membrane, and allowed the intracellular content of the bacterial cells to leak out through the membrane. Taken together, these results demonstrated that three peptides (KW-WK, FP-PF, and FW-WF) caused damage to the cytoplasmic membrane.

In conclusion, this study advocated an alternative approach to the design of AMPs according to the principles of symmetrical amino acid sequence. We used LFcinB18–28 as a template to synthesize four novel antimicrobial peptides with symmetrical amino acid sequences using the method of amino acid substitution. Two peptides, KW-WK and FP-PF, displaying high antimicrobial activity, low toxicity, and good stability had been devised successfully. These peptides might be useful as new antimicrobial drugs in the fields of food, medicine, and livestock feed. Although peptide FW-WF was relatively hemolytic, it still had good antimicrobial activity. Throughout the experiment, we observed the bacteriostatic mechanism for the three engineered peptides: KW-WK, FP-PF, and FW-WF. Unlike conventional antibiotics, which usually kill cells by inhibiting the synthesis of some substance in the cells, including cell wall, proteins, DNA or RNA, most AMPs share a cationic and amphipathic character that facilitates interaction with the anionic microbial membranes, leading to disruption of membrane integrity, permeabilizing microbial membranes, affecting the membrane potential, and resulting in cell death. The symmetrical amino acid sequence improved the antimicrobial activity to some extent, but the effect was closely related to the hydrophobicity, hydrophilicity, and charge of each AMP. Cumulatively, this work provides a theoretical basis for the design of more effective antimicrobial peptides.

## 4. Materials and Methods 

### 4.1. Materials

The peptides designed were synthesized and purified by GL Biochem (Shanghai, China) using matrix-assisted laser desorption/ionization time-of-flight mass spectrometry (MALDI-TOF MS, Linear Scientific Inc., Duquesne, PA, USA), a-cyano-4-hydroxycinnamic acid (HCCA) used as the matrix. The purity of the peptides was measured at over 95% by analytical reverse-phase high-performance liquid chromatography (LC 3000, Beijing, China). The molecular masses of the peptides were quantified using electrospray ionization mass spectrometry. The C-termini of all the peptides were amidated. The peptides were dissolved in deionized water at a concentration of 2.56 mM and then stored at −20 °C.

*E. coli* ATCC25922, *E. coli* UB1005, *Salmonella pullorum* C7913, *Salmonella enterica* subsp. CMCC 50071, *S. aureus* ATCC29213, *S.* *aureus* ATCC25923, *S. epidermidis* ATCC12228, *S. typhimurium* C7731, *S. typhimurium* ATCC14028, and human embryonic kidney 293 cells (HEK293) were obtained from the Harbin Veterinary Research Institute, CAAS (Harbin, China). An institutional review committee approved the using of human red blood cells in 21 March 2017, and written consent was obtained from the subjects. Mueller‒Hilton broth (MHB) and Mueller‒Hilton agar powder were purchased from AoBoX (Beijing, China). Dulbecco’s modified Eagle’s medium (DMEM) and fetal calf serum were purchased from Gibco (Beijing, China). All the chemicals and solvents were analytical grade unless otherwise noted and purchased from Kermel (Tianjin, China) and Sigma Chemical Company (Shanghai, China).

### 4.2. Design and Sequence Analysis of Peptides

An 11-residue peptide, LFcinB18–28, was a truncated peptide of LFcinB (amino acids 17–41). We transformed peptides LFcinB18–28 by replacing the charged amino acids at both ends of LFcinB18–28, and four engineered peptides were obtained. The amino acids used for the substitution included tryptophan (W), proline (P), phenylalanine (F), and the positively charged amino acids lysine (K) and arginine (R). 

The theoretical molecular weights (MW) and net charges of the peptides were calculated at http://web.expasy.org/compute_pi/. The hydrophobicity (H) and hydrophobic moment (μH) were calculated at http://heliquest.ipmc.cnrs.fr/.

### 4.3. Circular Dichroism (CD) Spectra

CD spectra were measured by a J-810 Spectropolarimeter (Jasco, Tokyo, Japan), which used a quartz cuvette with a 1.0 mm path length [32]. Next, 40 μM peptide samples were dissolved in 10 mM Phosphate Buffered Saline (PBS pH 7.4), 50% Trifluoroethanol (TFE), and 30 mM Sodium Dodecyl Sulfate (SDS). Spectra were monitored at 25 °C and recorded in the range of 190–250 nm at a scanning speed of 100 nm/min.

### 4.4. Antimicrobial Activity

The antimicrobial activity of the peptides were defined as the minimum inhibitory concentrations (MIC) according to the Clinical and Laboratory Standards Institute broth microdilution method as described previously [47]. Briefly, 50 μL two-fold serial dilutions of peptides (with final concentrations ranging from 0 μM to 256 μM) and 50 μL bacterial fluids diluted with MHB were added to 96-well microtiter plates. The plates were then incubated at 37 °C for 18 h. The MIC was defined as the minimum peptide concentration at which no bacterial growth was observed.

### 4.5. Hemolytic Activity

Hemolytic activity against human red blood cells (hRBCs) was determined as described before [48]. Briefly, 1 mL of fresh hRBCs was centrifuged in PBS (pH 7.2). After washing two times, the hRBCs were resuspended with PBS at a concentration of 1%. Next, 50 μL of the hRBCs solution was added to 50 μL PBS containing two-fold serial dilutions of peptides in each well. The plates were then incubated at 37 °C for 1 h and centrifuged at 1000× *g* for 5 min. The supernatants were removed, and the release of hemoglobin was determined by measuring the absorbance at 570 nm using a microplate reader (Bio-Rad, Hercules CA, USA). Erythrocyte suspensions in PBS and 0.1% Triton X-100 were used as negative and positive controls, respectively. Melittin was the control peptide.

### 4.6. Cytotoxicity

The cytotoxicity of each peptide was determined in HEK293 cells using the thiazolylblue (MTT) assay as described by Zhu [49]. Briefly, HEK293 cells were subcultured overnight in 96-well plates with 10% fetal calf serum and dimethyl sulfoxide (DMSO) at 37 °C in 5% CO_2_. The cell suspension was then prepared. Two-fold serial dilutions of the peptides were prepared using DMEM. Cell suspensions were added to the plates and incubated for 20–24 h at 37 °C in a cell incubator. Next, 40 μL MTT solution was added to every well, and the plates were incubated for 4 h at 37 °C. Finally, 150 μL DMSO was added to each well, the plates were shaken for 10 min, and the absorbance was measured at 492 nm using a microplate reader.

### 4.7. Stability

We assessed the salt stability, thermal stability, and enzymatic stability of the AMPs. Briefly, *E. coli* ATCC25922 was selected as the test bacterium [50]. The control group was untreated peptides. Different concentrations of salts (150 mM NaCl, 4.5 mM KCl, 6 μM NH_4_Cl, 1 mM MgCl_2_, 1 mM MgCl_2_, 8 mM ZnCl_3_, 2.5 mM CaCl_2_, and 4 mM FeCl_3_) were added to the incubation buffer to examine the effect of each salt on the antimicrobial activities of the peptides. AMPs were treated for 1 h at 100 °C to test the thermal stability. AMPs were also treated with trypsin, pepsin, papain, and protease K at a concentration of 1 mg/mL for 1 h at 37 °C to test enzyme stability.

### 4.8. Mechanism of Action of the Peptides

#### 4.8.1. Outer Membrane Permeability

The effect of AMPs on the outer cell membrane was determined using the outer membrane sensitive fluorescent dye Ethyleneimine (*N*-Phenyl-1-Naphthy Lamine, NPN) and *E. coli* UB1005 as previously described [41]. First, a bacterial suspension was prepared using 5 mM HEPES buffer (pH 7.4) containing 5 mM glucose. The cell suspension was then mixed with 1 mM NPN in a quartz cuvette, and the background fluorescence was recorded (excitation λ: 350 nm, emission λ: 420 nm). Peptides were added to the cuvette at different concentrations, and fluorescence was recorded until there was no additional increase in fluorescence. Polymyxin B (Sigma) was used as a positive control because of its strong outer membrane permeability properties.

The permeability of the outer membrane was calculated according to the following formula:

$$\mathrm{NPN}\left(\%\right)=\frac{F_{0bs}-F_{0}}{F_{100}-F_{0}}\cdot 100\%,$$

where *F*_0*bs*_ is the observed fluorescence at a given peptide concentration, *F*_0_ is the initial fluorescence of NPN in the absence of the peptide and *F*_100_ is the fluorescence of NPN upon addition of 10 μg/mL Polymyxin B.

#### 4.8.2. Inner Membrane Permeability

Changes in cell permeability were determined by measuring the intracellular activity of β-galactosidase in *E. coli* UB1005 using *O*-Nitrophenyl-β-d–galacto pyranoside (ONPG) as a substrate, as reported previously [39]. Briefly, *E. coli* UB 1005 grown to the logarithmic phase in MHB medium were centrifuged and re-suspended to an OD_600_ nm of 0.05 in PBS (5 mM, pH 7.4) with 2% lactose and 1.5 mM ONPG. Different concentrations of each peptide were added, and data were recorded every 2 min from 0 to 38 min at OD_420_.

#### 4.8.3. Cytoplasmic Membrane Depolarization

The effect of AMPs on the membrane potential of *E. coli* UB1005 was determined using the membrane potential-sensitive fluorescent dye diSC3-5, as described by Yang [18]. First, *E. coli* UB 1005 grown to the logarithmic phase were centrifuged and re-suspended to an OD_600_ nm of 0.05 in 5 mM of HEPES (pH 7.2) with 20 mM of glucose. Then, 0.4 mM diSC3-5 and 100 mM KCl were added, and 2 mL of the bacterial suspension with different concentrations of each peptide were placed in a cuvette. The fluorescence intensity was monitored at an excitation wavelength of 622 nm and emission wavelength of 670 nm from 0 to 300 s.

#### 4.8.4. Flow Cytometry

Flow cytometry can quantify the amount of cells with membrane damage as reported previously [51]. Briefly, *E. coli* ATCC 25922 grown to the logarithmic phase were centrifuged and re-suspended to an OD_600_ nm of 0.2 in PBS (pH 7.4). The suspensions were mixed with each peptide at 1/2× MIC or 1× MIC and incubated for 30 min at 37 °C with constant shaking (140 rpm). The cells were then harvested by centrifugation, washed three times with PBS, and incubated with Propidium Iodide (PI) at a final concentration of 10 mg/mL for 30 min at 4 °C. Finally, the unbound dye was removed by washing with an excess amount of PBS. The data were recorded using fluorescence-activated cell sorting (Becton Dickinson, Franklin Lakes NJ, USA).

#### 4.8.5. Electron Microscopic Studies

The effect of AMPs on bacterial cell integrity was characterized by scanning electron microscopy (SEM, Hitachi, Tokyo, Japan) and transmission electron microscopy (TEM, Hitachi, Japan) as described previously [52]. The bacterial cells (*E. coli* ATCC25922) grown to the logarithmic phase were treated with AMPs (1× MIC) for 2 h at 37 °C. For the SEM sample preparation, the cells were dehydrated and coated with gold-palladium, and then observed using S-4800 SEM. For the TEM sample preparation, the cells were dehydrated and incubated overnight in epoxy resin, and then cells were sectioned, stained, and observed using H-7650 TEM

### 4.9. Statistical Methods

In each experiment, triplicate samples were used. The data were subjected to one-way analysis of variance (ANOVA) to determine the significance of individual differences at *p* < 0.05 level. All statistical analyses were carried out using SPSS 20 statistical software package.

## Figures and Tables

**Table 1:** Peptide design and key physicochemical parameters.

Peptides	Sequence	Theoretical MW	Measured MW ^a^	Net Charge	μH ^b^	H ^c^	Purity
KW-WK	KWRRWQWRRWK-NH2	1772.1	1771.13	+7	0.441	0.251	96.94%
FP-PF	FPRRWQWRRPF-NH2	1631.9	1630.94	+5	0.159	0.478	97.58%
FW-WF	FWRRWQWRRRWF-NH2	1810.1	1809.70	+6	0.054	0.609	97.31%
KK-KK	KKRRWQWRRKK-NH2	1656.0	1655.05	+9	0.010	−0.338	95.21%
LFcinB18–28	KCRRWQWRMKK-NH2	1605.9	1605.02	+7	0.420	0.095	95.81%

^a^ Molecular weight (MW) was measured by mass spectroscopy (MS); ^b^ The hydrophobic moment (μH) of a peptide is its hydrophobic moment relative to that of a perfectly amphipathic peptide. This yields a better measure of peptide amphipathicity using different scales; ^c^ The mean hydrophobicity (H) is the total hydrophobicity (sum of all residue hydrophobicity indices) divided by the number of residues.

**Table 2:** Antimicrobial and hemolytic activities of the peptides.

	MIC ^a^ (μM)				
	KW-WK	FP-PF	FW-WF	KK-KK	LFcinB18–28
**Gram-negative bacteria**					
*E. coli* ATCC 25922	4	4	4	32	16
*E. coli* UB 1005	8	8	16	32	128
*Salmonella typhimurium* C 7731	16	64	16	>128	>128
*Salmonella typhimurium* ATCC 14028	32	32	128	>128	>128
*Salmonella pullorum* C 7913	16	16	8	128	>128
*Salmonella enterica* subsp. CMCC 50071	128	128	>128	128	>128
**Gram-positive bacteria**					
*S. aureus* ATCC 29213	8	32	>128	>128	>128
S. aureus ATCC 25923	32	32	16	128	128
*S. epidermidis* ATCC 12228	8	64	8	>128	>128
**MHC ^b^ (μM)**	>256	>256	8	>256	>256
**GM ^c^**	28.00	42.22	78.67	163.55	200.89
**TI ^d^**	9.14	6.06	0.10	1.56	1.28

The final concentrations of peptides ranged from 0 μM to 256 μM. ^a^ Minimum inhibitory concentrations (MIC) are defined as the lowest concentration of peptide that inhibits bacterial growth; ^b^ Minimum hemolytic concentration (MHC) is the lowest concentration of peptide that causes 5% hemolysis of human red blood cells (hRBCs); ^c^ GM denotes the geometric mean of MIC values from all microbial strains in this table; ^d^ Therapeutic index (TI) is the ratio of the MHC to the geometric mean of all MICs. Larger values indicate greater cell selectivity.

**Table 3:** MIC values of antimicrobial peptides in the presence of physiological salts.

Peptides	Na^+^	K^+^	Mg^2+^	Ca^2+^	Zn^2+^	Fe^3+^	NH_4_^+^	Control
KW-WK	8	4	8	8	8	4	8	4
FP-PF	64	8	8	8	8	4	8	4
FW-WF	8	4	4	8	4	4	2	4
KK-KK	16	16	64	16	8	8	8	32
LFcinB18–28	16	16	16	16	8	4	8	16

The concentrations of salts were 150 mM NaCl, 4.5 mM KCl, 6 μM NH_4_Cl, 1 mM MgCl_2_, 1 mM MgCl_2_, 8 mM ZnCl_3_, 2.5 mM CaCl_2_ and 4 mM FeCl_3_.

**Table 4:** MIC values of peptides following heating.

Peptides	100 °C	Control
KW-WK	4	4
FP-PF	8	4
FW-WF	8	4
KK-KK	>128	32
LFcinB18–28	16	16

The final concentrations of peptides ranged from 0 μM to 256 μM.

**Table 5:** MIC values of antimicrobial peptides in the presence of proteolytic enzymes.

Peptides	Trypsin	Pepsin	Papain	Protease K	Control
KW-WK	>128	16	8	8	4
FP-PF	128	16	8	4	4
FW-WF	>128	>128	4	16	4
KK-KK	>128	>128	>128	>128	32
LFcinB18–28	>128	>128	>128	>128	16

The final concentrations of peptides ranged from 0 μM to 256 μM.

## Abbreviations

AMPs	Antimicrobial Peptides
MIC	Minimum Inhibitory Concentration
hRBCs	Human Red Blood Cells
HEK293	Human Embryonic Kidney 293 Cell
GM	Geometric Mean
TI	Treatment Index
MW	Molecular Weights
PBS	Phosphate-Buffered Saline
TFE	Trifluoroethanol
SDS	Sodium Dodecyl Sulphate
NPN	*N*-Phenyl-1-Naphthy Lamine
PI	Propidium Iodide
